# Seafood allergy in children

**DOI:** 10.1186/2045-7022-1-S1-P36

**Published:** 2011-08-12

**Authors:** Paul Turner, Ian Ng, Andrew Kemp, Dianne Campbell

**Affiliations:** 1 Children's Hospital at Westmead, Allergy & Immunology, Westmead, NSW, Australia; 2 University of Sydney Medical School, Discipline of Paediatrics, Sydney, NSW, Australia

## Background

Both food allergy and seafood (fish, mollusc and crustacean) consumption have increased considerably over the past 40 years. There is limited published data on the epidemiology of seafood allergy in children.

## Methods

Using a retrospective chart review, we collected data on children presenting to our Tertiary Allergy Service with an allergic reaction to seafood between 2006 and 2009. Families were then contacted by postal questionnaire to assess the impact of the allergy on everyday life.

## Results

2999 children were seen during this period with a range of allergic problems, including food allergy. 167 (5.6%) had experienced a definite clinical reaction to seafood (103 male, 62%); 94% had evidence of co-existent atopic disease. The most common seafood implicated were: Prawn (26%), Unspecified white fish (10%), Tuna (8%), Salmon (8%). In 14%, the exact type of fish could not be recalled by the parent or identified by the physician.

21% had experienced anaphylaxis to seafood. Over 50% of crustacean-allergic children could tolerate fish. However, cross-sensitization was very common in fish-allergic children, with one third reporting clinical reactions to at least two species of fish; 59% were sensitized to crustacean while 22% had clinical allergy to crustacean. 16% developed symptoms to fish vapours. In children with allergy to tuna and/or salmon, at least 21% were able to tolerate the fish in a tinned form.

119 families received the questionnaire, of whom 94 responded (79%). Parents of children with a history of anaphylaxis were to be more compliant with dietary advice. Parents of fish-allergic children tended to be more cautious, taking care to avoid foods labeled 'traces' despite medical advice to the contrary.

## Conclusion

This study demonstrates that seafood is a common and important cause of food allergy in Australian children, and highlights the high rate of anaphylaxis in this population.

